# Cerebellar hemorrhage in patients treated with edoxaban for portal vein thrombosis after hepatobiliary surgery: a report of two cases

**DOI:** 10.1186/s40792-020-01086-3

**Published:** 2020-12-10

**Authors:** Hiroya Iida, Toru Miyake, Masaji Tani, Takuya Tanaka, Kayo Kawakami, Yoshihiro Ikuno, Ryoichi Mandai, Tomoharu Shimizu

**Affiliations:** 1 Department of Surgery, Shiga University of Medical Science, Otsu, Shiga 520-2192 Japan; 2 Medical Safety Section, Shiga University of Medical Science Hospital, Otsu, Shiga 520-2192 Japan

**Keywords:** Intracerebral breeding, Cerebral hemorrhage, Direct oral anticoagulants, DOACS

## Abstract

**Background:**

The standard therapeutic agent administered for portal vein thrombosis (PVT) in patients with or without cirrhosis is warfarin or low-molecular weight heparin. However, therapy with edoxaban appears to be one of the most promising treatments for patients who require anticoagulation therapy. We encountered two cases of cerebellar hemorrhage in patients treated with edoxaban for PVT after hepatobiliary surgery during the past 2 years.

**Case presentation:**

*Case 1* A 67-year-old male underwent cholecystectomy and choledocholithotomy with choledochoduodenostomy to treat choledocholithiasis after cholangitis. Enhanced computed tomography (CT) on the 1st postoperative day (POD) revealed thrombosis in the left and anterior segment of the portal vein branches. We administered antithrombin III concentrate with heparin for 5 days; thereafter, we switched to 60 mg edoxaban. A sudden decrease in the patient’s level of consciousness was observed due to cerebellar hemorrhage on POD 27. Cerebellar hemorrhage was successfully treated with craniotomy hematoma evacuation and ventricular drainage; however, the patient died from aggravation of hepatic failure due to PVT and intra-abdominal infection. *Case 2* A 67-year-old male received laparoscopic microwave coagulation therapy for two hepatic nodules suggestive of hepatocellular carcinoma in the left lobe of the liver due to alcoholic hepatitis. Enhanced CT on POD 5 revealed a thrombosis in the 4th segment branch of the portal vein, and the patient was treated with 60 mg edoxaban. Cerebellar hemorrhage with ventricular perforation occurred on POD 15. Cerebellar hemorrhage was successfully treated by craniotomy hematoma evacuation with ventricular drainage. Prolonged consciousness disorder persisted, and the patient was transferred to another medical facility for rehabilitation 49 days after brain surgery.

**Conclusions:**

Although edoxaban is recently described to be one of the options for patients with PVT who require anticoagulation therapy instead of heparin or warfarin, it should be used with caution, given its propensity to induce severe hemorrhagic adverse events in cases such as those described above. The monitoring of hepatic dysfunction and decision for continuation of drug may be required during edoxaban use for PVT, especially after hepatobiliary surgery.

## Background

Portal vein thrombosis (PVT) often develops after abdominal surgery, especially in patients with cirrhosis, or those who have undergone hepatobiliary and pancreatic surgery. Vitamin K antagonists, warfarin, or low-molecular weight heparin are the standard treatments for PVT in patients with or without cirrhosis [1]. Direct oral anticoagulants (DOACs) are indicated for the prevention of stroke and systemic embolism in adult patients with nonvalvular atrial fibrillation (NVAF), for the treatment of venous thromboembolism (VTE), and for the prevention of recurrent VTE in adults; however, portal vein thrombosis is not officially indicated. There is a growing body of evidence regarding the safety and efficacy of DOACs for the treatment of VTE [2]. We encountered two cases of cerebellar hemorrhage in patients treated with edoxaban for PVT after hepatobiliary surgery during the past 2 years.

## Case presentation

*Case 1* A 67-year-old male (height, 170.0 cm; weight, 65.9 kg) was referred to our hospital for treatment of choledocholithiasis after cholangitis. He had a medical history of cerebral infarction and gastrostomy, with Billroth-II re-constriction due to gastric ulcer 27 years ago. The patient received medications including amlodipine, azilsartan, and clopidogrel. Preoperative laboratory examination showed slight elevation in aspartate aminotransferase (53 U/L) and alanine transaminase (64 U/L) levels, and Child–Pugh A score of 5 points. The patient subsequently underwent cholecystectomy and choledocholithotomy with choledochoduodenostomy. There was no injury or additional manipulation of portal vein system during surgery for choledocholithiasis.

**Fig. 1:**
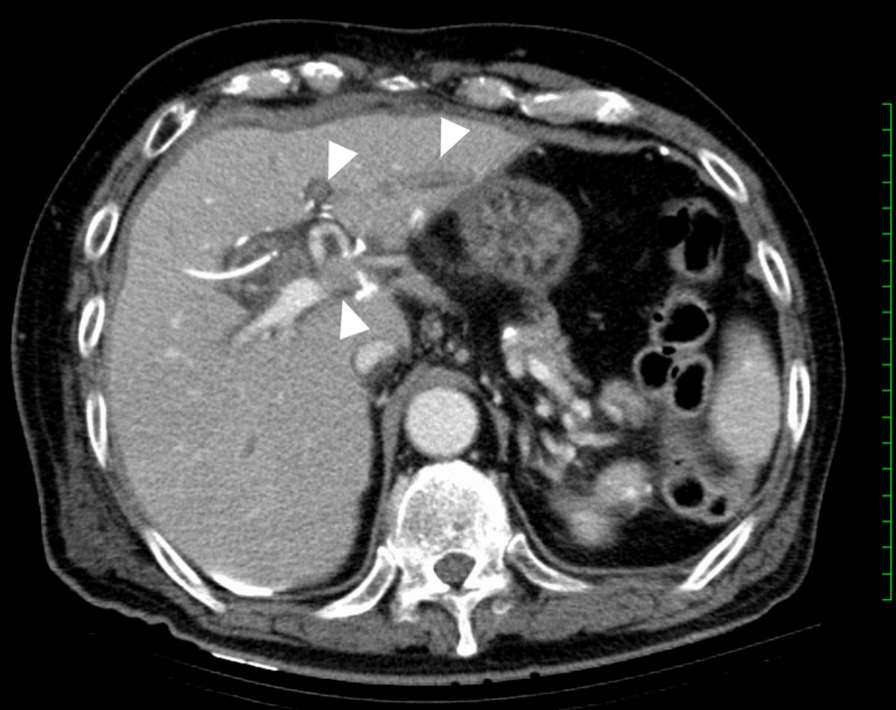
Abdominal computed tomography (CT) images of case 1. An enhanced abdominal CT scan showed thrombosis in the left and anterior segment branches of the portal vein (white triangle) on POD 1

**Fig. 2:**
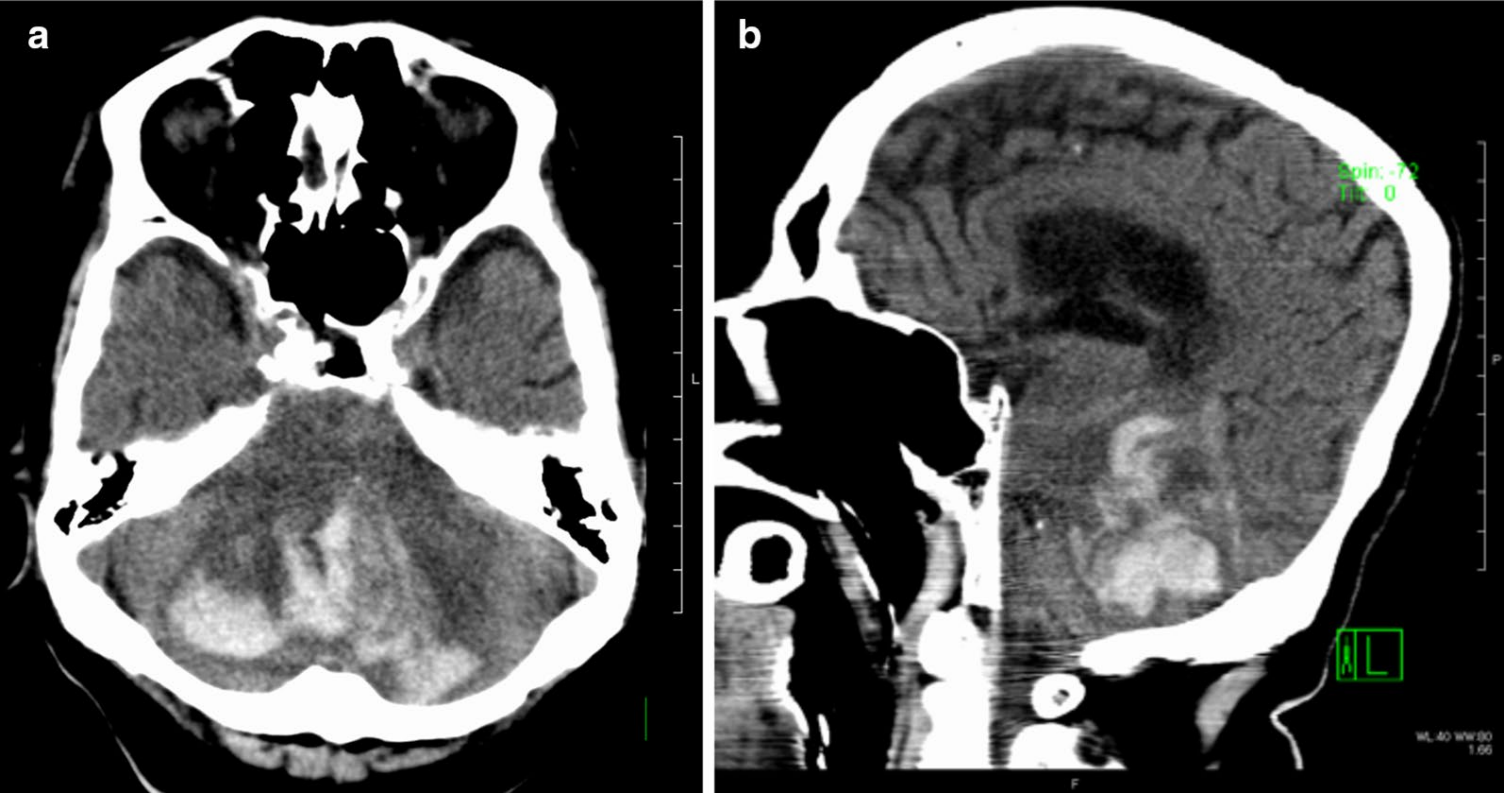
Brain computed tomography (CT) images of case 1. Brain CT revealed cerebellar bleeding on POD 27

Enhanced computed tomography (CT) on the first postoperative day (POD) revealed thrombosis at the left and anterior segment of the portal vein branches (Fig. 1). D-dimer was elevated up to 64.0 µg/mL and systemic heparinization was started as a result. There was no significant change in PVT as confirmed by an additional CT examination on POD 7. Antithrombin III concentrate was administered for 5 days; thereafter, we switched from heparin to clopidogrel (75 mg) for cerebral infarction and edoxaban (60 mg) for PVT. Anastomotic leakage of the choledochoduodenostomy was observed, and percutaneous drainage of the intra-abdominal abscess was performed on POD 14. A sudden decrease in the patient’s level of consciousness was observed due to cerebellar hemorrhage on POD 27 (Fig. 2). Laboratory examination on POD 27 revealed the following: platelet count (PLTS), 196,000/µL; prothrombin time activity (PT-ACT), 52%; fibrinogen (Fib), 646 mg/dL; D-dimer, 5.5 µg/mL; prothrombin time international normalized ratio (PT-INR), 1.4; albumin (ALB), 2.5 mg/dL; total-bilirubin (T-Bil), 7.94 mg/dL; creatinine (Cre), 0.53 mg/dL; estimated glomerular filtration rate (eGFR), 116.2 mL/min/1.73 m^2^; C-reactive protein (CRP), 10.16 mg/dL. Cerebellar hemorrhage was successfully treated with craniotomy hematoma evacuation and ventricular drainage; however, the patient died from aggravation of hepatic failure due to PVT and intra-abdominal infection on POD 51.

**Fig. 3:**
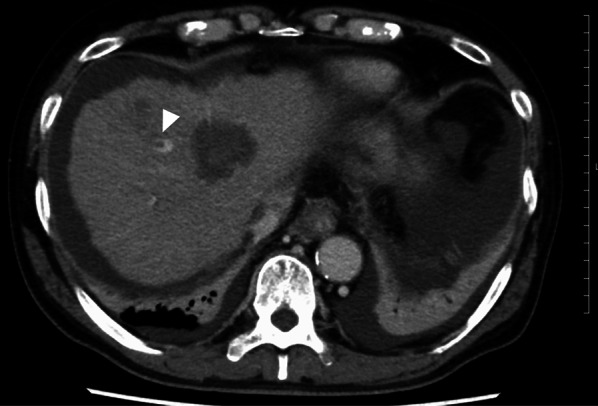
Abdominal computed tomography (CT) images of case 2. An enhanced abdominal CT scan revealed thrombosis in the branches of the portal vein (white triangle) on POD 5

**Fig. 4:**
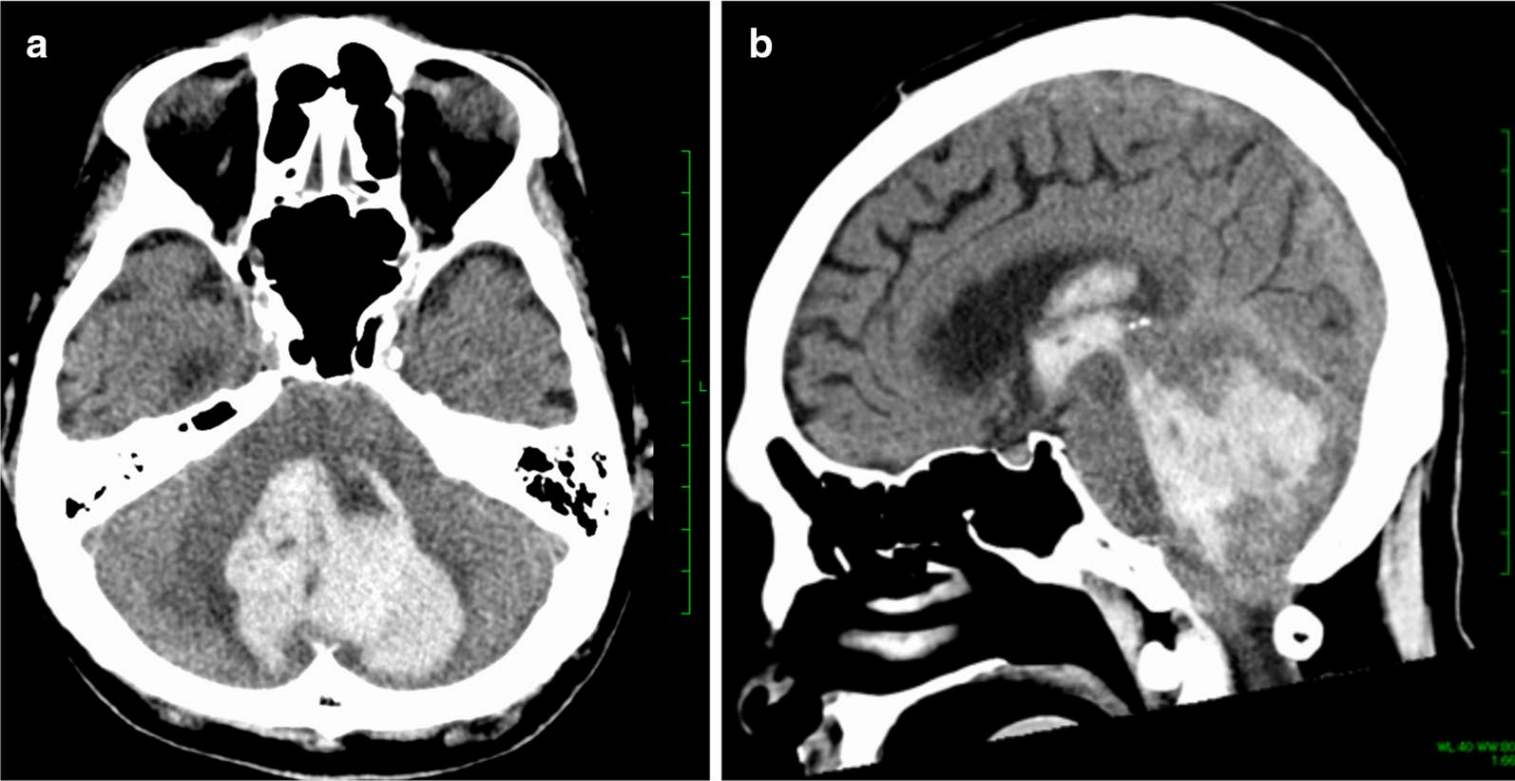
Brain computed tomography (CT) images of case 2. Cerebellar bleeding with intraventricular rupture was observed on POD 17

*Case 2* A 67-year-old male (height, 173.6 cm; weight, 65.5 kg) was admitted for treatment of two hepatic nodules (16 mm at S2/4 and 12 mm at S2) suggestive of hepatocellular carcinoma in the left lobe of liver due to alcoholic hepatitis (T2, N0, M0, Stage II). Preoperative laboratory examination showed the following: PLTS, 109,000/µL; PT-ACT, 74%; PT-INR, 1.14; Fib, 203 mg/dL; D-dimer, 0.9 µg/mL; ALB, 3.8 mg/dL; T-Bil, 1.29 mg/dL; Cre, 0.9 mg/dL; eGFR, 65.1 mL/min/1.73 m^2^; CRP, 0.11 mg/dL; indocyanine green retention rate at 15 min (ICGR15), 45.7%; and a Child–Pugh A score of 6 points. Consequently, laparoscopic microwave coagulation therapy was planned for this patient. Since PVT and congestion of portal blood flow were observed in the left and 4th segment branch of the portal vein after microwave coagulation therapy by endoscopic ultrasonography during surgery, administration of antithrombin III concentrate with enoxaparin was administered for 5 days after surgery. Enhanced CT on POD 5 revealed a thrombosis in the segment 4th branch of the portal vein (Fig. 3), and the patient was administered with 60 mg edoxaban as a result. Consciousness disorder occurred on POD 15, and CT examination revealed cerebellar hemorrhage with ventricular perforation (Fig. 4). Blood examination at this time revealed the following: PLTS, 181,000/µL; PT-ACT, 52%; PT-INR, 1.39; Fib, 224 mg/dL; D-dimer, 5.6 µg/mL; ALB, 3.7 mg/dL; T-Bil, 1.23 mg/dL; Cre, 1.23 mg/dL; eGFR, 88.5 mL/min/1.73 m^2^. Although cerebellar hemorrhage was successfully treated by craniotomy hematoma evacuation with ventricular drainage, the consciousness disorder persisted. The patient was transferred to another medical facility for rehabilitation 49 days after brain surgery.

## Discussion

DOACs have a favorable risk–benefit profile, with significant reductions in stroke, intracranial hemorrhage, and mortality, and with similar major bleeding events as with warfarin; however, their use results in increased gastrointestinal bleeding in patients with NVAF [3]. Edoxaban is indicated for the prevention of stroke and systemic embolism in adult patients with NVAF, in the treatment of VTE, and for the prevention of recurrent VTE in adults worldwide.

Warfarin or low-molecular weight heparin are standard therapeutic agents for PVT in patients with cirrhosis [1]. Recently, Nagaoki et al. demonstrated that edoxaban following danaparoid sodium is an effective anticoagulant and may be considered as an alternative to warfarin for PVT in patients with cirrhosis. In this study, grade 3 or 4 adverse events (gastrointestinal bleeding) were observed in 10% of all patients (3/20 in the edoxaban group and 2/30 in the warfarin group). No gastrointestinal bleeding or other side effects were observed in patients with Child–Pugh B or C liver disease, and no cerebral bleeding was reported in this study population [4]. Moreover, recent review articles have shown that DOACs are safe and efficacious alternatives to traditional anticoagulants with low-molecular weight heparin and warfarin in the treatment of acute PVT with or without cirrhosis [2]. We also employed edoxaban as an alternative to warfarin for PVT during the past 2 years. The indications for the use of edoxaban for PVT in our hospital are main trunk thrombus and first to third branch thrombus regardless of side of liver lobe. Additionally, superior mesenteric vein and splenic vein thrombus are also indicated the drug administration. However, patients of tumor thrombus and acute complete obstruction of the main trunk of portal vein by thrombus are not indicated for edoxaban administration.

The decision of dose reduction in edoxaban for patients with PVT is considered to be as same as adaptive diseases. The recommended dose of edoxaban was 60 mg once daily, while a 30 mg dose once daily is recommended in patients with one or more of the following clinical factors: moderate or severe renal impairment (creatinine clearance, 15–50 mL/min), low body weight (≤ 60 kg), and concomitant use of P-glycoprotein inhibitors, such as ciclosporin, dronedarone, erythromycin, or ketoconazole. No dose reduction is required in the elderly population. Especially in hepatic function, patients with elevated liver enzymes (alanine aminotransferase (ALT) or aspartate transaminase (AST) > 2 × upper limit of normal (ULN)) or total bilirubin ≥ 1.5 × ULN, were excluded in clinical studies [5, 6]. The use of edoxaban in patients with moderate or severe hepatic impairment (Child–Pugh B and C) is not recommended, as these patients may have intrinsic coagulation abnormalities. No dose reduction is required in patients with mild hepatic impairment (Child–Pugh A) [7].

Absorption of edoxaban occurs predominantly in the proximal small intestine. Surgeries such as Roux-en-Y gastric bypass could reduce the absorption of edoxaban because of distal segments of the gastrointestinal tract with low edoxaban absorption capability [8]. The decrease in absorption capability of edoxaban was estimated in case 1 due to the history of gastrostomy with Billroth-II re-constriction. A 60 mg dose of edoxaban was administered to our patients without clinical risk factors, such as low body weight and renal and hepatic impairment.

The concomitant use of antiplatelet drugs (aspirin, cilostazol, dipyridamole and clopidogrel) may increase the risk of bleeding events. Edoxaban should be used with caution considering risk and benefit for patients with the concomitant use of these drugs. In ENGAGE AF-TIMI 48, concomitant use of clopidogrel monotherapy was permitted and resulted in increased clinically relevant bleeding [5]. Recent study demonstrated that the safety of edoxaban in combination with clopidogrel in patients with atrial fibrillation who had percutaneous coronary intervention. There was no significant difference in bleeding and stroke events edoxaban combination compared to warfarin combination [9]. We considered that doublet anticoagulants use (edoxaban and clopidogrel) could benefit the patient with systemic arteriosclerosis to prevent recurrent cerebral infarction in case 1.

Postoperative jaundice due to surgical complications was observed in case 1. Renal clearance accounts for approximately 50% of the administered dose, while metabolism and biliary/intestinal excretion account for the remaining clearance [10]. Therefore, cholestasis due to anastomotic leakage of the choledochoduodenostomy appeared to be influenced the excretion of edoxaban from bile system in case 1; therefore, the monitoring of hepatic dysfunction is required for patients with PVT during edoxaban administration.

The ICG retention test has become a safe, rapid, reproducible, inexpensive and noninvasive tool for the assessment of liver function. Clinical evidence suggests that the ICG retention test can enable the establishment of tailored management strategies by providing prognostic information; however, it is not commonly used worldwide because of its low levels of evidences [11]. In case 2, subclinical hepatic impairment seemed to progress by ICGR15 otherwise preoperative Child–Pugh A, and hepatic resection was avoided according to this result. We decided edoxaban use and dose according to liver enzymes, total bilirubin and Child–Pugh A, since there is no evidence to reduce edoxaban dose according to the results of ICGR15.

A phase 3 study involving 8292 patients with acute VTE (the Hokusai-VTE) demonstrated that once-daily edoxaban was as effective as warfarin for the prevention of recurrent symptomatic VTE and was associated with a significantly lower rate of bleeding. Clinically relevant bleeding (major or non-major) occurred in 349 of 4118 patients (8.5%) in the edoxaban group and in 423 of 4122 patients (10.3%) in the warfarin group (hazard ratio [HR] 0.81; 95% confidence interval [CI] 0.71 to 0.94; *P* = 0.004). Major bleeding, including cerebral bleeding, occurred in 56 patients (1.4%) in the edoxaban group and 66 patients (1.6%) in the warfarin group (HR 0.84; 95% CI 0.59 to 1.21) [6].

Cerebellar hemorrhage occurred in 2 of the 13 PVT cases treated with edoxaban in our department during the past 2 years. The incidence of cerebral bleeding seemed to be high compared to those reported in previous large-scale studies treated with edoxaban [6]. Very few studies have investigated the efficacy and safety of edoxaban administration for patients with PVT who have received recent abdominal surgery. Edoxaban should be used with caution, and proper informed consent should be obtained due to the potential of severe hemorrhagic adverse events after hepatobiliary surgery. We continued the use of edoxaban in other patients who have received its administration safely until the occurrence of case 2 checking hepatic and renal function during administration. We stopped the new entry of off-label use of edoxaban for PVT after the occurrence of case 2. A large-scale clinical study is required to clarify the feasibility and safety of edoxaban in patients with PVT who have received recent abdominal surgery.

## Conclusions

Although the standard treatment for PVT in patients with or without cirrhosis is warfarin or low-molecular weight heparin, edoxaban use is recently described to be an option for patients with PVT who require anticoagulation therapy. We encountered two cases of cerebellar hemorrhage in patients treated with edoxaban for PVT after hepatobiliary surgery over a relatively short period of time. Edoxaban should be used with caution for patients with PVT due to the propensity for severe hemorrhagic adverse events observed in our cases. The monitoring of hepatic dysfunction and decision for continuation of drug may be required during edoxaban use for PVT, especially after hepatobiliary surgery.

## Data Availability

Data sharing is not applicable to this article as no datasets were generated or analyzed during the current study.

## Abbreviations

PVT: Portal vein thrombosis
DOACs: Direct oral anticoagulants
NVAF: Nonvalvular atrial fibrillation
VTE: Venous thromboembolism
POD: Postoperative day
PLTS: Platelet count
PT-ACT: Prothrombin time activity
PT-INR: Prothrombin time international normalized ratio
Fib: Fibrinogen
T-bil: Total-bilirubin
ALB: Albumin
Cre: Creatinine
eGFR: Estimated glomerular filtration rate
CRP: C-reactive protein
ICG: Indocyanine green
